# Evaluation of a functionalized chitosan and alginate multilayer conformal nanocoating toward improving islet survival in syngeneic mouse islet transplantation

**DOI:** 10.1002/btm2.70039

**Published:** 2026-01-21

**Authors:** Michael Yilma Yitayew, Alexandre Bay, Ling Li, Ciriaco A. Piccirillo, Maryam Tabrizian

**Affiliations:** ^1^ Department of Biomedical Engineering McGill University Montreal Quebec Canada; ^2^ Department of Microbiology and Immunology McGill University Québec Montreal Canada; ^3^ Program of Infectious Diseases and Immunity in Global Health, Centre for Translation Biology (CTB) The Research Institute of the McGill University Health Centre (RI‐MUHC) Montreal Quebec Canada; ^4^ Centre of Excellence in Translational Immunology (CETI), McGill University Montreal Quebec Canada; ^5^ Department of Anatomy and Cell Biology McGill University Montreal Quebec Canada; ^6^ Faculty of Dental Medicine and Oral Health Sciences McGill University Montreal Quebec Canada

**Keywords:** conformal coating, islet transplantation, layer‐by‐layer, nano encapsulation, T1D

## Abstract

Encapsulation of pancreatic islet transplants with nano‐thin conformal coatings has been reported to maintain islet cell function and minimize immune rejection in type 1 diabetes (T1D) treatment. Our work investigated a novel combination of non‐immunogenic polyelectrolytes, tetrahydropyran triazole phenyl‐alginate (TZ‐AL) and quaternized phosphocholine‐chitosan (PC‐QCH), for layer‐by‐layer self‐assembly onto the surface of mouse islets. Building on previous work validating coating characteristics and biocompatibility using cell‐derived spheroids, we assessed the immunoprotective properties of the polyelectrolyte coating. This was done through in vitro co‐culture of the polyelectrolytes with mouse‐derived splenocytes enriched for antigen‐presenting cells (APCs) and syngeneic transplantation of coated mouse islets into STZ‐induced diabetic mice. Results indicated that the polyelectrolytes may downregulate APC activation and maturation in vitro. In addition, coated islets successfully restored normoglycemia in syngeneic transplants, as demonstrated by blood glucose measurements, intraperitoneal glucose tolerance tests, and graft immunostaining. These results suggest that the polyelectrolyte coating may modulate APC activation and that coated islets exhibit therapeutic efficacy for glycemic control in T1D.

**Translational Impact Statement**

This study demonstrates a significant advancement in type 1 diabetes (T1D) cell therapy by showing that PC‐QCH/TZ‐AL nanocoated mouse islets can restore normoglycemia for 30 days in diabetic C57BL/6 mice. The layer‐by‐layer nanoencapsulation provides both protection and sustained islet function, highlighting its excellent biocompatibility and validating its therapeutic potential. The results underscore its promise for application in human islet transplantation and support its progression toward clinical translation for T1D treatment.

## 1 INTRODUCTION

Type 1 diabetes (T1D) is a chronic autoimmune disease that destroys insulin‐producing pancreatic beta cells, resulting in severe hyperglycemia and other health complications.^1^ Beta cell replacement therapies, such as allogenic islet transplantation, offer a long‐term treatment option for a subset of patients unresponsive to exogenous insulin injections, the clinical gold standard.^2^ However, allogeneic islet transplantation requires systemic immunosuppression, which can cause organ toxicity and increase susceptibility to infections. As a result, bioengineering approaches seek alternative methods to protect islet grafts from immune‐mediated rejection.^3^ Among these approaches, conformal coating represents an ideal configuration, offering minimal capsule thickness for improved circulation and islet function while providing an immunoprotective barrier against the host immune system.^4^

Layer‐by‐layer (LbL) deposition of biocompatible polyelectrolytes produces a multilayer conformal coating with several advantages, including the ability to optimize thickness and coverage by changing the number of layers.^5^ It can also serve as a depot for the targeted local release of growth factors, drugs, or other molecules to improve graft survival.^5^, ^6^, ^7^ Many polyelectrolyte pairs have been investigated, with a trend in the field toward optimizing polyelectrolyte immunomodulatory capacity.^8^ Following this rationale, we previously conceptualized and characterized a polyelectrolyte pair based on modifications of natural and biocompatible chitosan and alginate polymers.^9^ Phosphocholine (PC), a zwitterionic side‐group, was chosen to enhance chitosan's immunomodulatory properties, as PC is implicated in pathogen immune evasion. While the mechanisms of PC‐modified macromolecule immune evasion are unclear, one hypothesis suggests their potential role in preventing activation of toll‐like receptors (TLRs).^10^ They may also induce IL‐10 production in B cells and suppress the production of pro‐inflammatory cytokines like TNF‐α, IL‐6, and IL‐12.^11^ PC‐chitosan was further modified with quaternary amine groups, creating a mild polycation (PC‐QCH) as a building block of a LbL self‐assembled nanocoating.

The use of tetrahydropyran triazole phenyl‐alginate (TZ‐AL) as a polyanion enables multilayer nanocoating self‐assembly via electrostatic pairing with PC‐QCH and the islet surface, further enhancing the coating's immunomodulatory effect. Triazole derivatives exhibit diverse pharmacological activity, notably as an anti‐fibrotic and anti‐inflammatory moiety for biomedical applications.^12^, ^13^, ^14^, ^15^ The addition of a tetrahydropyran triazole phenyl amine side‐group to alginate was demonstrated to reduce fibrosis and innate immune cell attachment (macrophages and neutrophils) on the material in mouse studies.^14^ Structurally similar molecules have also been shown to decrease human T‐cell proliferation and inhibit cytokine expression by reducing NFAT1 (nuclear activator of T‐cells) nuclear localization and activity.^12^ Although the anti‐fibrotic effect of triazole‐modified macromolecules is established, the underlying cellular mechanisms, especially in innate immune cells, warrant further investigation. Thus, studying the interaction of PC‐QCH and TZ‐AL with immune cells is crucial to elucidating the role of PC and TZ in innate immune cell activation and modulation.

This study investigated the role of the aformentioned polyelectrolyte pair in immune cell activation and whether the nanocoating of primary islets maintains in vivo function and graft survival in syngeneic transplant models using immunocompetent mice. In vitro analysis of splenocyte stimulation with the polyelectrolytes in solution and coated polystyrene particles was performed to explore the interaction between these polymers and innate immune cells, focusing on antigen‐presenting cells (APCs). APCs were chosen for this in vitro model because their activation and maturation are critical for antigen processing and presentation, initiating and driving both innate and adaptive immune signaling toward implanted biomaterials.^16^, ^17^ Furthermore, prior work demonstrated in vitro biocompatibility and in vivo graft function in diabetic immunodeficient mice using mouse MIN6 cell‐derived spheroids. Building on these findings, this study assessed the graft function and fate of coated mouse islets transplanted into immunocompetent mice. Streptozotocin‐induced diabetic C57BL/6 (B6) mice were transplanted with coated syngeneic (B6) islets, and graft function was evaluated by periodic blood glucose measurements and intraperitoneal glucose tolerance test (IPGTT) over 30 days. In addition, post‐sacrifice histology and immunostaining were conducted after 30 days to assess islet graft persistence, endocrine hormone expression, and neovascularization.

## 2 MATERIALS AND METHODS

### 2.1 Reagents

L‐α‐glyceryl‐phosphocholine was purchased from Biosynth (San Diego, California). Chitosan (100–300 kDa) was purchased from MP Biomedical (Solon, Ohio). Alginate (250–350 kDa) was purchased from Sigma Aldrich (Oakville, Ontario). trans‐N,N′‐Dimethylcyclohexane‐1,2‐diamine and 2‐chloro‐4,6‐dimethoxy‐1,3,5‐triazine (CDMT) were purchased from TCI Chemicals. Methanol, acetone, dichloromethane, and acetonitrile were purchased from Fisher Scientific. All other chemicals used in this study were purchased from Sigma Aldrich (Oakville, Ontario). All chemicals were used as received.

RPMI 1640 medium, fetal bovine serum (FBS), penicillin/streptomycin, Hank's balanced salt solution (HBSS), and phosphate‐buffered saline (PBS) were all purchased from Gibco through Fisher Scientific. BSA Fraction V was purchased from Roche. Mouse insulin ELISA (Mercodia) was purchased from Cedarlane, and mouse C‐peptide ELISA was purchased from ALPCO. alamarBlue reagent (Invitrogen) was purchased from Thermo Scientific. Only ultra‐pure deionized (mQ) water (18.2 MΩ.cm) from a Barnstead nano‐pure filtration system was used for experiments.

### 2.2 Synthesis of quaternized phosphocholine chitosan (PC‐QCH)

PC‐QCH was synthesized based on a previously published protocol.^9^ Briefly, L‐α‐glyceryl‐phosphocholine (4.0 g in 120 mL of water) was cooled on an ice bath, and sodium periodate (6.8 g) was added. The mixture was stirred for 5 h, and the water was removed under reduced pressure. The resulting solids were dispersed in methanol, stirred, and filtered to obtain a clear solution, which was concentrated under reduced pressure to yield the product, phosphocholine aldehyde (PC‐AL). Chitosan (2.0 g) was dissolved in 80 mL of 2% acetic acid, and 3.0 mmol of PC‐AL dissolved in 24 mL of methanol was slowly added into the chitosan mixture. The mixture was stirred for 10 min, and the pH was balanced to 6.50 by slowly adding 10 M NaOH. After stirring for 1 h, sodium cyanoborohydride (15 mmol in 10 mL water) was added slowly into the reaction, and the mixture was stirred overnight. The solution was titrated to pH 11, filtered to remove non‐soluble material, and purified by dialysis for 5 days (mQ water – 2 days, 50 mM NaOH – 1 day, mQ water – 2 days) before freeze‐drying to obtain the final product, phosphocholine chitosan (PC‐CH). 1.0 g of PC‐CH formulation was mixed with dimethyl sulfate (19.2 mL), water (4.0 mL), sodium hydroxide (1.44 g), and sodium chloride (1.052 g) and stirred at room temperature overnight. The resulting mixture was purified by dialysis for 3 days in 0.2 M NaCl and eventually mQ water, filtered to remove undissolved polyelectrolyte, and freeze‐dried to obtain the final product, PC‐QCH.

### 2.3 Synthesis of tetrahydropyran triazole phenyl alginate (TZ‐AL)

TZ‐AL was synthesized based on a previously published protocol.^9^ A mixture of sodium azide (254.7 mg), sodium ascorbate (70.4 mg), trans‐N,N′‐Dimethylcyclohexane‐1,2‐diamine (140 μL), and copper (I) iodide (169.71 mg) was prepared in methanol (3.75 mL). Tetrahydro‐2‐(2‐propynyloxy)‐2H‐pyran (0.5 mL) and 4‐iodobenzyl amine (1 g) were then added, and the reaction was stirred overnight at 55°C with a reflux condenser set up. The crude product was purified using silica column chromatography with 3% NH_4_OH in methanol:dichloromethane (10%–20% v/v). The pure amine product was resuspended in acetonitrile and coupled with alginate by dissolving 300 mg of alginate in 8.1 mL of water and adding 131.7 mg of CDMT (0.5 equiv.) and 165 μL of NMM (1 equiv.). A total of 5.4 mL of amine (1.52 mmol) in acetonitrile was added dropwise into the reaction and stirred overnight at 55°C. The crude product was diluted in mQ water and filtered to remove insoluble material. The resulting product was purified by dialysis in mQ water for 2 days and freeze‐dried to obtain the final product, tetrahydropyran triazole phenyl alginate (TZ‐AL). FITC‐labeled alginate was also produced using this exact same coupling reaction with fluorescein‐amine (43 mg, 0.1 equiv) and alginate (500 mg) in the dark.

### 2.4 Mouse islet isolation

All studies were conducted after ethical approval and in accordance with the institutional ethical committee of McGill University (AUP# MCGL‐8238) and the Canadian Council of Animal Care. Male C57BL/6J mice aged 8–10 weeks old were purchased from Jackson Laboratories and maintained in pathogen‐free conditions for the duration of the study.

Mouse islets were isolated and purified based on previously established protocols.^18^, ^19^, ^20^ Mice were euthanized using isoflurane/CO_2_ inhalation followed by cervical dislocation. The common bile duct was tied at the ampulla of Vater using a suture, and 2–3 mL of cold collagenase solution (1 mg/mL in HBSS) was slowly injected into the common bile duct. Once the pancreas was distended, it was dissected from the surrounding tissue and kept on ice, immersed in collagenase solution. Once dissections were complete, each pancreas was digested at 37°C, and enzymatic activity was quenched after 20 min of incubation with 20% FBS solution in HBSS. The islets were resuspended in HBSS with 0.1% BSA and filtered through a 500 μm sieve to remove undigested tissue, followed by density gradient centrifugation with Histopaque‐1077 and complete RPMI media. Afterward, islets were cultured at 37°C, 5% CO_2_ using complete RPMI media containing 10% FBS and 1% penicillin/streptomycin.

### 2.5 Multilayer nanocoating

PC‐QCH (10 mg/mL) was dissolved overnight in HBSS, pH balanced to 7.4 using 0.1 M NaOH and sterile filtered using a 0.22 μm PES filter inside a biosafety cabinet (BSC) before coating. TZ‐AL (20 mg/mL) was dissolved overnight in 0.8% saline and diluted in HBSS to 3 mg/mL, pH balanced to 7.4 with 0.1 M HCl or NaOH and sterile filtered using a 0.22 μm PES filter inside a BSC. All polyelectrolytes formed clear, transparent solutions in buffer.

#### 2.5.1 Multilayer coating of polystyrene microspheres

Multilayer nanofilms were self‐assembled on the surface of polystyrene microspheres (20.00 μm Polybead® carboxylate microspheres, Polysciences) following previously established protocols with some modifications.^21^, ^22^ Microspheres were washed by centrifuging at 400 *g* for 1 min, and 1 mL of polycation solution (PC‐QCH, 10 mg/mL in HBSS) was added and mixed for 20 min on a rotator. This was followed by aspiration and washing steps (2× in HBSS) to remove excess polyelectrolyte, and the process was repeated with 1 mL of polyanion solution (TZ‐AL, 3 mg/mL in HBSS). This cycle was repeated until the formation of 4 bilayers of PC‐QCH/TZ‐AL, and microspheres were suspended in HBSS. Coating deposition was confirmed with zeta potential analysis using a Brookhaven Zeta PALS instrument. Ten runs were conducted per measurement at 5 cycles/run, and each measurement was performed in triplicate. In addition, FITC‐alginate was used to confirm coating deposition using confocal microscopy. Images were taken on a Zeiss LSM710 microscope with the equipped Zen software and later processed using ImageJ.

#### 2.5.2 Multilayer coating of primary islets

**FIGURE 1:**
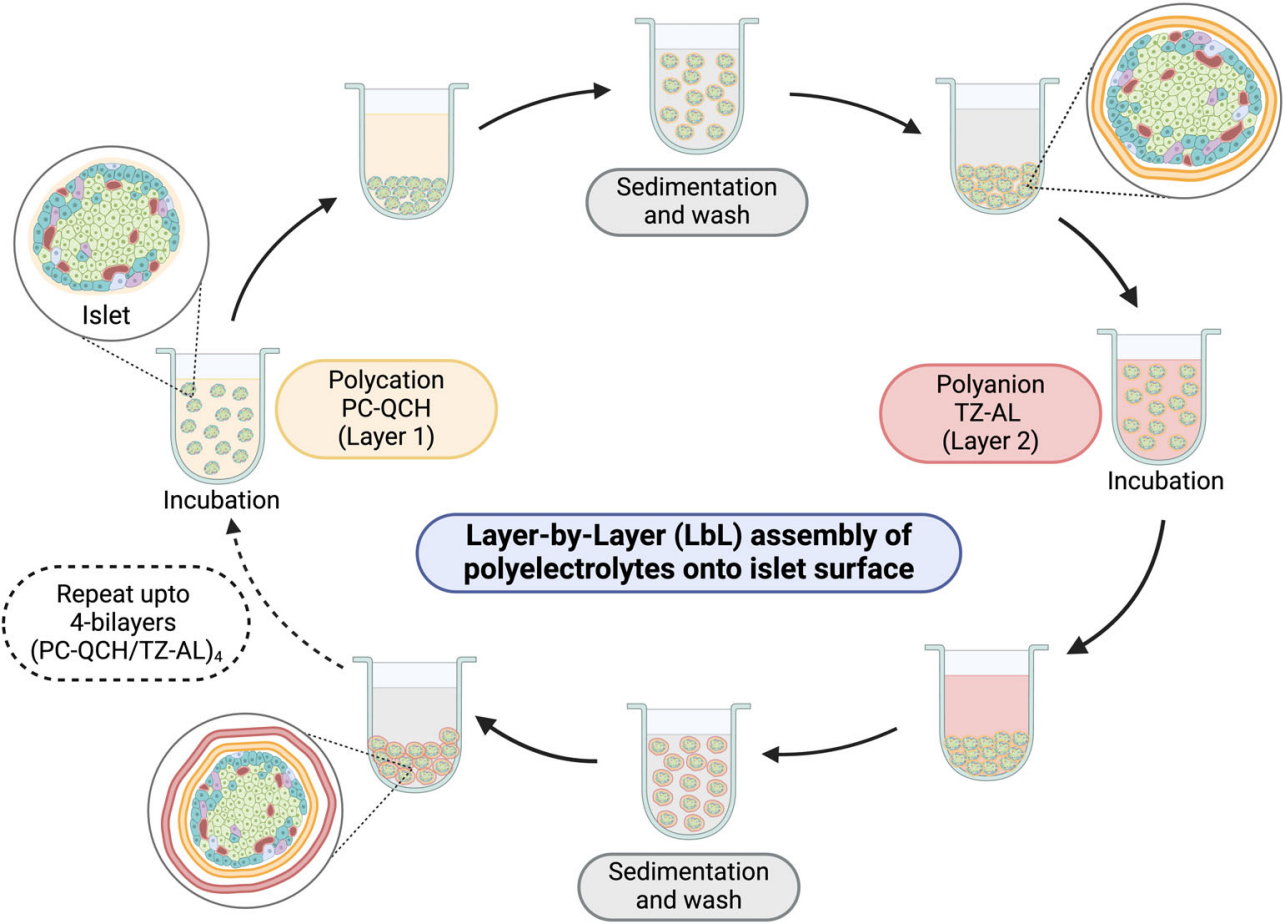
Schematic representation of pancreatic islet conformal coating using layer‐by‐layer self assembly of PC‐QCH/TZ‐AL polyelectrolytes.

Multilayer nanofilms were self‐assembled on the surface of primary mouse islets via sequential incubation of the islets in solutions of the proposed polyelectrolytes, as shown in Figure 1. Islets were washed (2× in HBSS) in 12‐well plates, and 1 mL of polycation solution (PC‐QCH, 10 mg/mL in HBSS) was added and incubated for 10 min, followed by aspiration and washing steps (2× in HBSS) to remove excess polyelectrolyte. The process was repeated with 1 mL of polyanion solution (TZ‐AL, 3 mg/mL in HBSS), and this cycle was repeated until the formation of 4 bilayers. At the end of the coating procedure, the buffer was replaced by culture media (complete RPMI), and islets were cultured overnight at 37°C, 5% CO_2_. Coating deposition was confirmed using confocal microscopy by including FITC‐alginate as part of the coating along with Hoechst 33342 staining to localize the cells in relation to the coating.

### 2.6 Splenocyte isolation

Splenocytes and inguinal lymph nodes were isolated from male C57BL/6 mice following previously established protocols.^23^ Briefly, mice were euthanized using isoflurane/CO_2_ inhalation followed by cervical dislocation. The spleen and inguinal lymph nodes were dissected and transferred to a sterile tube containing cRPMI 1640 supplemented with 10% fetal bovine serum, 1% penicillin/streptomycin, and 50 μM 2‐mercaptoethanol and kept on ice until processing inside a biosafety hood (BSC). Spleens were perfused with medium and pressed through a 70 μm nylon mesh cell strainer using the back of a sterile syringe plunger. The cell suspension was then centrifuged at 400 g for 5 min, and RBCs were lysed upon resuspension in ACK lysis buffer for 30 s. The lysis buffer was quenched with PBS, and cells were centrifuged at 400 g for 5 min, with the pellet then resuspended in PBS and filtered through a 70 μm nylon mesh into a new sterile 15 mL Falcon tube. The resulting suspension was quenched once more with PBS and centrifuged at 400 g for 5 min. The cell pellet was resuspended in cRPMI, and viable cells were counted with a hemocytometer by Trypan blue exclusion.

T cells were depleted from the cell suspension for in vitro activation assays by staining with anti‐mouse CD3e‐APC Monoclonal Antibody (Cat. #17‐0031‐82, eBioscience, 2.5 μL/100 μL) and incubating for 20 min at 4°C away from light. Stained cells were centrifuged at 400 g for 5 min, and the resulting pellet was resuspended in 1 mL PBS, stained with 100 μL Anti‐APC MicroBeads (Cat. #130‐090‐855, Miltenyi Biotec), and incubated for 20 min at 4°C away from light. A total of 500 μL PBS was added following incubation, and unlabeled CD3^−^ cells were isolated using the “DEPLETES” program on an AutoMACS Pro Separator (Miltenyi Biotec). The resulting suspension was quenched with PBS, centrifuged at 400 g for 5 min, and resuspended in cRPMI before counting viable cells via Trypan blue exclusion with a hemocytometer.

### 2.7 In vitro activation of antigen presenting cells

Cells were resuspended to a concentration of 1 × 10^6^ cells/mL, with 100 μL being administered to each experimental well in flat‐bottom 96‐well plates. 4× stock mixes of polymer and LPS were prepared, and 50 μL of each mix was added to their respective wells. Wells not receiving polymers, LPS, or either were given 50 μL of cRPMI to replace the respective mix. Plates were then incubated at 37°C, 5% CO_2_ for 24 h before extracellular antibody staining. For microspheres, spheres were initially suspended at a stock concentration of 3.9 × 10^6^ particles/mL in a volume of 270 μL. The stock was diluted with cRPMI to 1.7 × 10^6^ particles/mL, with 50 μL of spheres added to each well (8.5 × 10^4^ particles/well). T cell‐depleted splenocytes were diluted to 1.7 × 10^6^ cells/mL, with 100 μL of cells added to each well (1.7 × 10^5^ cells/well).

After 24 h, cells were resuspended and transferred to 96‐well V‐bottom plates for staining. Following a primary resuspension and centrifugation step (400 *g*, 5 min), the flat‐bottom plates were washed with cold PBS to ensure maximal pickup of cells, then transferred to the same V‐bottom plates and centrifuged again. Pellets were briefly vortexed to break them up before they were treated with 50 μL Fc receptor block (1:100, BD Biosciences # 553141) mixed with PBS. Cells were incubated for 20 min at 4°C away from light, then quenched with 200 μL PBS and centrifuged as before. An extracellular antibody mix was prepared in PBS using the following antibodies (all from BD Biosciences unless otherwise specified): anti‐mouse CD19‐BUV737 (1:100, #612781), anti‐mouse F4/80‐PE‐Cy7 (1:100, eBioscience #25‐4801‐82), anti‐mouse CD11c‐PE (1:100, eBioscience #12‐0114‐82), anti‐mouse I‐A^b^ (1:100, eBioscience #553551), anti‐mouse CD86‐BUV395 (1:100, #564199), and SYTOX Blue (1:1000, Invitrogen #S34857). Following the quench and centrifugation steps after Fc block, pellets were briefly vortexed and stained with 50 μL of extracellular antibody mix, then incubated for 20 min at 4°C away from light. Cells were quenched with PBS, centrifuged as before, then resuspended to a final volume of 150 μL before acquisition on the LSRFortessa X‐20 (BD Biosciences) with the gating strategy for analysis shown in Figure S1.

### 2.8 Characterization of conformal coating on primary islets

FITC‐alginate and Hoechst 33342 staining were used to confirm the deposition of coating using confocal microscopy. FITC‐alginate (3 mg/mL) was used in the coating steps, and islets were fixed with 4% paraformaldehyde and stained with Hoechst 33342 (20 μg/mL). Images were taken on a Zeiss LSM710 microscope with the equipped Zen software and later processed using ImageJ.

Cell viability and metabolic activity of coated mouse islets were measured using the alamarBlue assay (Invitrogen). One hundred islets were placed in 24‐well plates, and their media was replaced with 300 μL of media containing 1× alamarBlue Reagent in the dark. The plates were incubated in the dark for 4 h, and absorbance measurements were taken using a Spectramax i3 multimode spectrometer at 570 and 600 nm. The result values are presented as percent reduction, which is calculated with a formula provided by the kit manufacturer. Glucose‐stimulated insulin secretion (GSIS) assay was performed in Krebs buffer containing HEPES (25 mM), NaCl (115 mM), NaHCO_3_ (24 mM), KCl (5 mM), MgCl_2_ (1 mM), BSA (0.1%), and CaCl_2_ (2.5 mM) at pH 7.4, adjusted using 1 M NaOH or 1 M HCl. Mouse islets (coated or non‐coated; 50 islets per well) were pre‐conditioned in a 12‐well plate at 37°C for 1 h in 1 mL of low‐glucose (2.8 mM) Krebs buffer. The solution was then aspirated and replaced with 0.5 mL of fresh low‐glucose (2.8 mM) Krebs buffer, followed by a 1 h incubation. The supernatant was collected for analysis, and 0.5 mL of high‐glucose (16.7 mM) Krebs buffer was added to each well. After a 1 h incubation period, the supernatant was collected for analysis. Insulin concentrations in the supernatants were quantified using a mouse insulin ELISA kit (Mercodia) according to the manufacturer's instructions.

### 2.9 Diabetes induction and islet transplantation

C57BL/6 mice were rendered diabetic with an intraperitoneal injection (200 mg/kg) of streptozotocin (STZ) (BioShop Canada, Burlington, Ontario) reconstituted fresh in citrate buffer (50 mM, pH 4.5). Non‐fasting blood glucose levels were measured from tail vein blood periodically using a commercial glucose meter (Freestyle Lite, Abbott). Diabetes onset was established with two consecutive BG measurements above 16.7 mM (300 mg/dL). Mice were transplanted with 500 coated or non‐coated islets, and experimental groups were randomly assigned. Mice were prepared for surgery and anesthetized with 2% isoflurane inhalant. The kidney was exposed through a dorsal incision, and islets were slowly infused using a sterile PE‐50 tubing and a Hamilton syringe into a pouch generated under the kidney capsule with a blunt capillary. Incisions were closed with a surgical suture and skin clips. Mice were monitored for blood glucose and weight. An intraperitoneal glucose tolerance test (IPGTT) was performed following previously established methods.^24^ Mice were fasted for 6 h before intraperitoneal injection of glucose solution (2 g/kg), and blood glucose measurements were taken from the tail vein as before. Serum C‐peptide concentrations were quantified using a mouse C‐peptide ELISA (ALPCO, Salem, NH) following the instructions provided by the kit manufacturer.

### 2.10 Histological and immunostaining assessment

The kidneys of euthanized mice were explanted and fixed in 10% formalin before paraffin embedding. Tissue sections (5 μm) were mounted on glass slides and deparaffinized/rehydrated with sequential immersion in Citrisolv (3×), Citrisolv/Ethanol (1:1), and a series of ethanol solutions (100% to 30% ethanol in water), followed by immersion in distilled water. For histological analysis, rehydrated samples were stained with Hematoxylin and Eosin solutions, mounted, and imaged with a light microscope. For immunostaining, rehydrated slides were immersed in citrate buffer (10 mM, pH 6) and incubated at 90–100°C for 30 min for antigen retrieval. Afterward, the tissue was incubated in blocking buffer containing tris‐buffered saline (TBS) with 0.1% Triton‐X and 5% bovine serum albumin (BSA) for 1 h at room temperature. Sections were then incubated with rabbit anti‐insulin antibody (1:500, Abcam ab181547), rabbit anti‐glucagon antibody (1:100, Cell Signaling Technologies #2760), or rabbit anti‐CD31/PECAM‐1 antibody (1:50, Sino Biological, #10148‐R324) overnight at 4°C. The slides were washed with TBS + 0.05% Tween‐20 and incubated with Alexa Fluor 647‐labeled goat anti‐rabbit IgG secondary antibody (1:100, Thermo Fisher Scientific A21244) for 1 h at room temperature. After washing with TBS + 0.05% Tween‐20, sections were mounted using Vectashield® mounting medium containing the nuclei stain DAPI. Images were taken on a Zeiss LSM710 microscope with the equipped Zen software and later processed using ImageJ.

### 2.11 Statistical analysis

Data are represented as mean ± standard deviation (SD), and *n* = 3 unless stated otherwise. ANOVA (one‐way or two‐way depending on data set) and Tukey's multiple comparison tests were utilized to compare more than two means, and an unpaired two‐tailed *t*‐test with Welch's correction was utilized to compare data sets with only two means. The data were considered statistically significant when *p* < 0.05 (* *p* ≤ 0.05, ** *p* ≤ 0.01, *** *p* ≤ 0.001, **** *p* ≤ 0.0001). All graphs and statistical analyses were performed using GraphPad Prism.

## 3 RESULTS

### 3.1 Activation of APCs is attenuated in the presence of PC‐QCH and TZ‐AL

**FIGURE 2:**
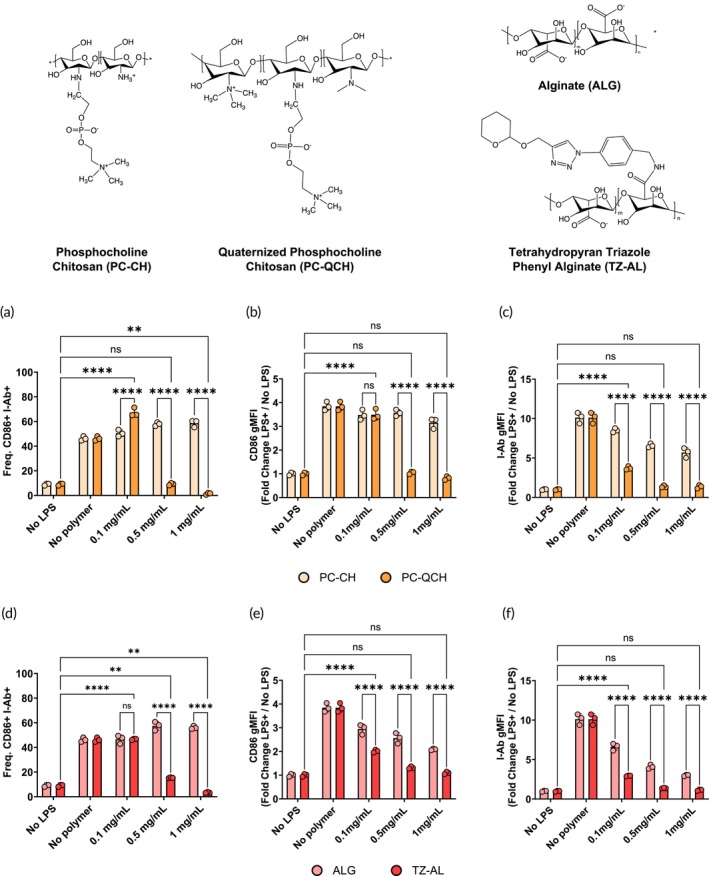
APC stimulation with LPS (1 μg/mL) and different concentrations of PC‐CH vs. PC‐QCH (a–c) and ALG versus TZ‐AL (d–f), followed by flow cytometry analysis of CD86 and I‐Ab+ cell frequency (a,d). No polymer (1 μg/mL LPS) and no LPS groups were used as control. gMFI of CD86 (b, e) and I‐Ab (c, f) are represented as fold change relative to cells with no LPS. Data are represented as mean ± SD. ns = *p* > 0.05, **p ≤ 0.01, ****p ≤ 0.0001, two‐way ANOVA with Tukey's test.

APC activation and maturation are significant events in the signaling cascade leading to the recognition of foreign antigens by the host immune system. Consequently, biomaterials that reduce this activation may modulate the immune response to transplants favorably and improve transplant performance. In this study, flow cytometric analysis of APC stimulation with PC‐QCH resulted in a decreased frequency of activated APCs relative to PC‐CH or cells alone at all LPS doses (Figures 2a and S2). This reduction was particularly evident at higher polymer concentrations (0.5 and 1 mg/mL), even without LPS present (Figure S2). At high PC‐QCH concentrations (0.5 and 1 mg/mL), the frequency of activated APCs was similar to or lower than that of non‐activated (no LPS) controls, regardless of LPS dose. Given the pro‐inflammatory nature of LPS, increasing LPS concentration generally increased APC activation frequency, as expected. Similarly, TZ‐AL showed a decreased frequency of activated APCs relative to alginate or cells alone at all LPS doses (Figures 2d and S3), with marked effects at higher polymer concentrations (0.5 and 1 mg/mL), even without LPS present (Figure S3).

In addition to cell frequency, geometric mean fluorescence intensity (gMFI) was analyzed to further assess the level of cell activation in each group. The results mirrored the cell frequency data, with PC‐QCH reducing CD86 (Figure 2b) and I‐Ab (Figure 2c) levels relative to non‐activated cells (no LPS). Notably, higher polymer concentrations (0.5 mg/mL and 1 mg/mL) reduced APC activation at all LPS doses to levels comparable to non‐activated cells (no LPS) (Figures 2b,c and S2). Similarly, TZ‐AL reduced CD86 and I‐Ab levels relative to non‐activated cells (no LPS), with significant effects at higher polymer concentrations (0.5 and 1 mg/mL). These concentrations reduced APC activation at all LPS doses to levels comparable to non‐activated cells (no LPS) (Figures 2e,f and S3). Thus, both PC‐QCH and TZ‐AL attenuated LPS‐mediated APC activation in vitro.

### 3.2 APC activation is attenuated by coated polystyrene microspheres

**FIGURE 3:**
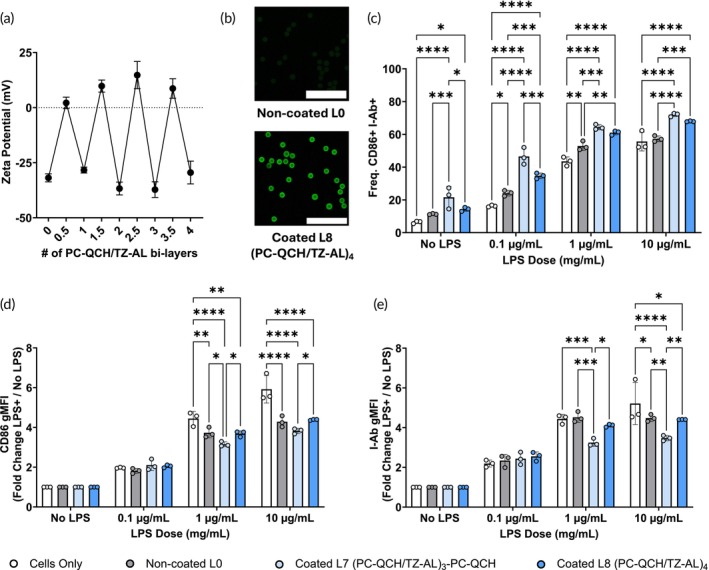
Zeta potential analysis of coating deposition on microsphere surface for 4 bilayers of PC‐QCH/TZ‐AL (L8) (a). Confocal microscopy images of coated microspheres containing FITC‐labeled alginate (green) (b). Scale bar = 150 μm. APC stimulation with LPS and non‐coated (L0) or coated microspheres (L7 – PC‐QCH outer layer or L8 – TZ‐AL outer layer) followed by flow cytometry analysis of CD86 and I‐Ab+ cell frequency (c). gMFI of CD86 (d) and I‐Ab (e) are represented as fold change relative to cells with no LPS. Data are represented as mean ± SD. ns = *p* > 0.05, *p ≤ 0.05, **p ≤ 0.01, ***p ≤ 0.001, ****p ≤ 0.0001, two‐way ANOVA with Tukey's test.

Since the polyelectrolytes used for the proposed coating reduced APC activation in vitro, their effect on APCs was evaluated when presented as a surface coating. Coated microspheres were used to isolate the interaction of the coating material, eliminating confounding factors associated with co‐culture approaches using coated cell spheroids or islets. Zeta potential analysis confirmed cyclical changes in surface charge during coating, from negative on the bare microsphere to positive after PC‐QCH deposition, and back to negative after TZ‐AL deposition (Figure 3a). In addition, confocal microscopy using FITC‐labeled alginate further confirmed bilayer deposition (Figure 3b). Co‐culture with T‐cell‐depleted splenocytes showed a significant increase in the frequency of activated APCs on coated microspheres. However, comparing gMFI of CD86 and I‐Ab revealed decreased activation levels (Figure 3c–e). While this decrease was dose‐dependent on LPS quantity, similar patterns were observed without LPS (Figure 3c). Microspheres with a PC‐QCH outer layer (L7) showed significantly reduced APC activation by gMFI analysis of CD86 (Figure 3d) and I‐Ab (Figure 3e). On the other hand, microspheres with a TZ‐AL outer layer (L8) showed activation levels similar to non‐coated microspheres (L0). These results demonstrate the coating's beneficial role in reducing APC activation and potential inflammation.

### 3.3 Coated mouse islets maintain their viability and insulin secretion in vitro

**FIGURE 4:**
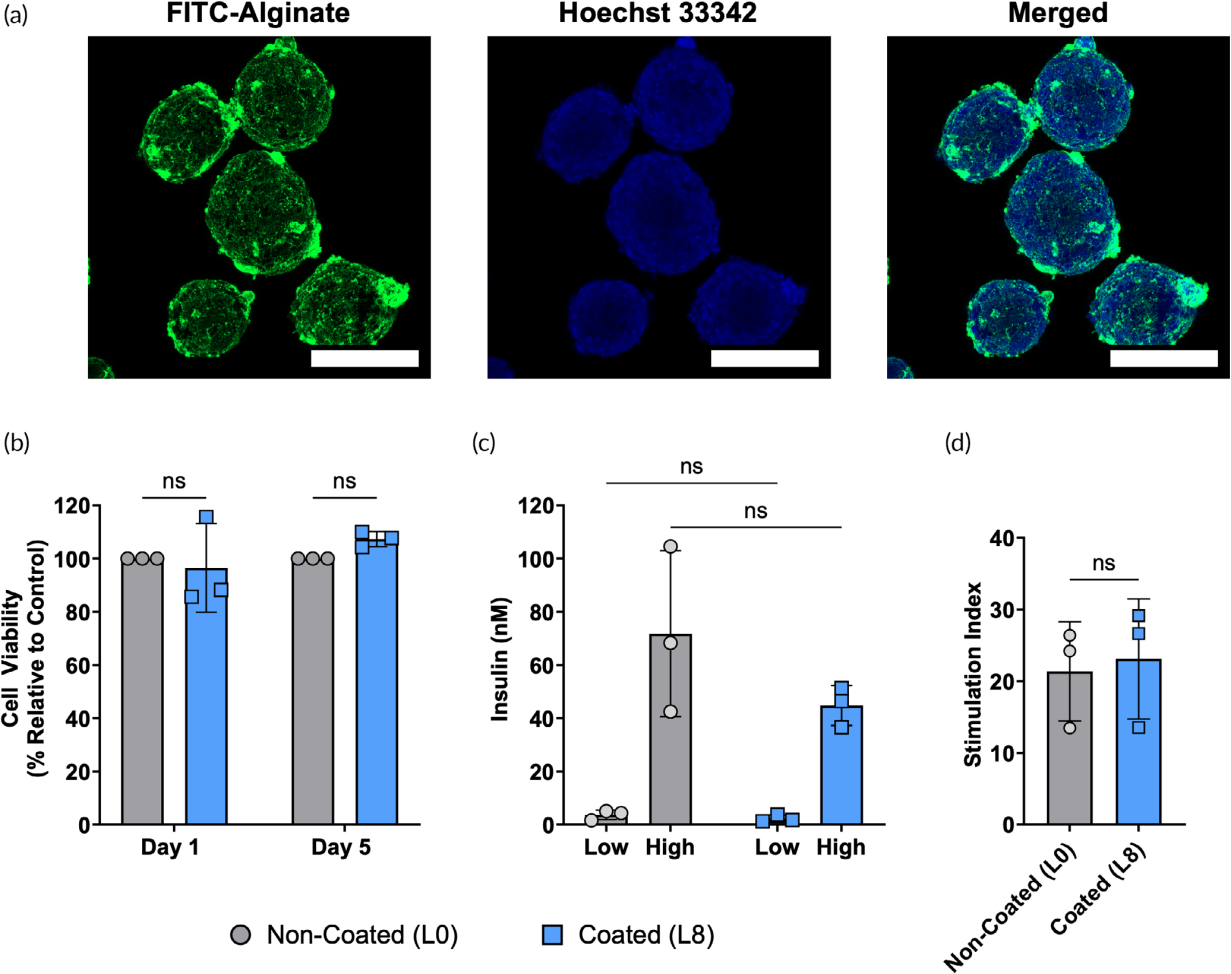
Confocal microscopy images of coated mouse islets with FITC‐labeled alginate (green) and Hoechst 33342 nuclear dye (blue) (a). Scale bar = 200 μm. Cell viability of coated and non‐coated mouse islets measured at day 1 and day 5 using the alamarBlue metabolic assay (b). Glucose‐stimulated insulin secretion (GSIS) of coated and non‐coated mouse islets at low (2.8 mM) and high (16.7 mM) glucose concentrations (c). Stimulation index indicating fold change in secretion at 16.7 mM glucose from 2.8 mM glucose (d). Data are represented as mean ± SD. ns = *p* > 0.05, *p ≤ 0.05, two‐way ANOVA with Tukey's test.

Confocal microscopy with FITC‐labeled alginate confirmed coating formation on mouse islets (Figures 4a and S4), revealing uniform coating morphology consistent with previous work on mouse‐derived beta cell spheroids.^9^ Coated islets maintained viability for up to 5 days in vitro, comparable to non‐coated islets, as shown by the alamarBlue metabolic assay (Figure 4b). Importantly, glucose‐stimulated insulin secretion (GSIS) assays demonstrated comparable insulin secretion between coated and non‐coated islets (Figure 4c). The stimulation index (fold change in secretion at 16.7 mM glucose relative to 2.8 mM glucose) remained consistent between both groups (Figure 4d). This validation was crucial before transplanting coated islets into diabetic mice to assess in vivo function.

### 3.4 Coated mouse islets can restore normoglycemia in syngeneic transplant models

**FIGURE 5:**
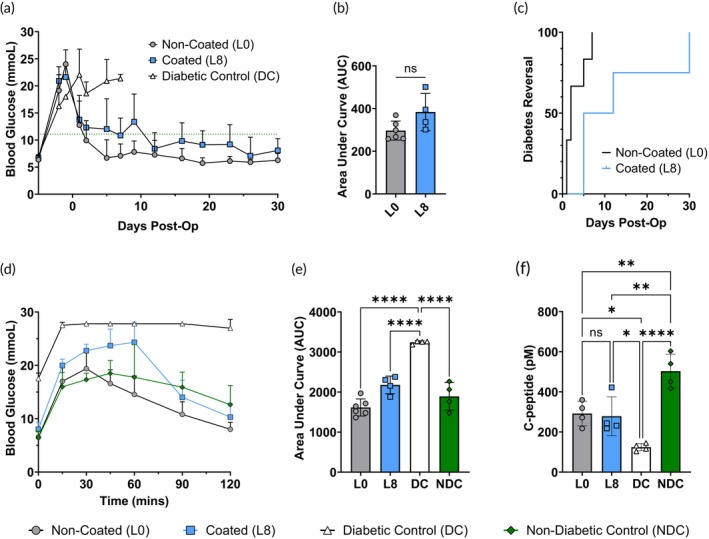
Average non‐fasting blood glucose levels of diabetic B6 mice after kidney sub‐capsular transplantation of non‐coated (*n* = 6) and coated (*n* = 4) mouse islets (a). The green horizontal line marks the threshold for normoglycemia (11.1 mM). Area under curve calculations (AUC) (b) and diabetes reversal rate for mice that received coated and non‐coated islets (c). Intraperitoneal glucose tolerance test (IPGTT) 30 days after kidney sub‐capsular transplantation (*n* = 6 non‐coated, *n* = 4 coated, *n* = 4 diabetic control (DC), and *n* = 4 non‐diabetic control (NDC)) (d) and IPGTT AUC (e). Fasting serum c‐peptide measured at 30 days after transplantation (*n* = 4 all groups) (f). Data are represented as mean ± SD. ns = *p* > 0.05, *p ≤ 0.05, **p ≤ 0.01, ***p ≤ 0.001, ****p ≤ 0.0001.

Islets isolated from B6 mice and coated with 4 bilayers (L8) of PC‐QCH and TZ‐AL restored normoglycemia (BG < 11.1 mM) for 30 days when transplanted into STZ‐induced diabetic B6 mice (Figure 5a). Transplanted mice showed marked differences in blood glucose levels compared to diabetic controls (DC). Area under the curve (AUC) analysis showed no significant difference in blood glucose levels between mice receiving coated islets (L8) and those receiving non‐coated islets (L0) (Figure 5b). Although delayed in the coated group, diabetes reversal was achieved in 100% of mice in both groups (Figure 5c).

An intraperitoneal glucose tolerance test (IPGTT) 30 days post‐transplant demonstrated comparable islet function between mice receiving coated and non‐coated islets (Figures 5d and S5). IPGTT curves for transplanted mice (coated or non‐coated) and non‐diabetic controls (NDC) all showed the expected immediate rise in blood glucose concentrations within 15–30 min after IP glucose injection, followed by a decline in concentration after 1 h. This was a clear indication of glucose‐dependent insulin production from islet grafts and resembled the expected reaction based on existing knowledge of carbohydrate metabolism.^25^ These results were also comparable to NDC mice, as confirmed by AUC analysis (Figure 5e), with no significant differences between transplanted mice and NDC mice. As expected, diabetic mice showed no response to IPGTT, exhibiting a statistically significant increase in IPGTT AUC compared to all other groups. Fasting serum C‐peptide analysis revealed reduced levels in transplant recipients compared to NDC mice, but no significant differences were observed between mice receiving coated and non‐coated islets (Figure 5f). Furthermore, transplanted mice exhibited higher C‐peptide concentrations than diabetic controls (DC), corroborating IPGTT blood glucose results.

**FIGURE 6:**
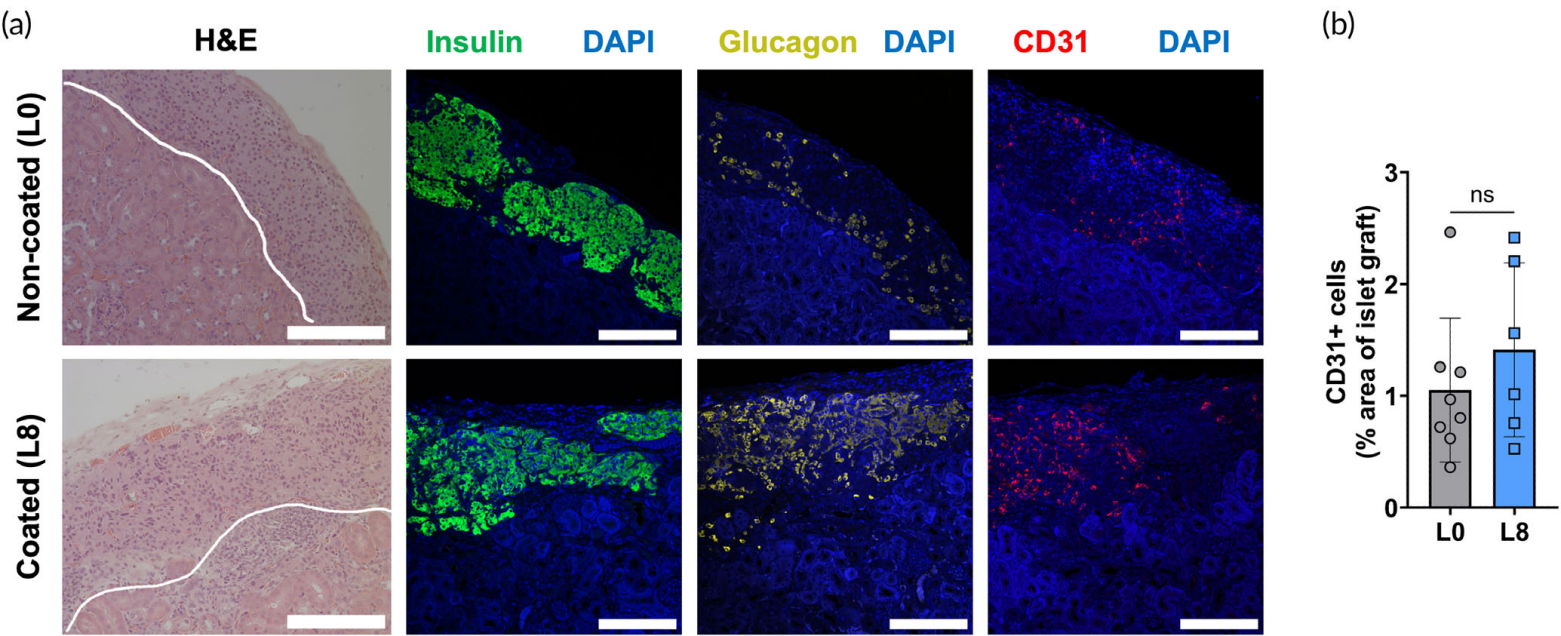
Post‐sacrifice histological H&E staining and immunostaining for insulin (green), glucagon (yellow), CD31 (red), and cell nuclei (blue) to assess tissue architecture of kidneys receiving non‐coated or coated islets (a). The white line in H&E images indicates the border between islets (above) and kidney cells (below). Scale bar = 200 μm. Quantitative image analysis of CD31‐positive cells within islet grafts (b). Data are represented as mean ± SD. ns = *p* > 0.05.

Histological analysis (H&E staining) of kidneys retrieved 30 days post‐transplant showed coated islets persisting in the kidney capsule (Figure 6a). Islet incorporation into kidney tissue and remodeling of the subcapsular space were evident, with distinct morphological differences between kidney cells and islets. Insulin and glucagon staining confirmed the presence of beta and alpha cells, respectively, and sustained graft secretory function (Figure 6a). As expected, given the relative abundance of beta cells in mouse islets, insulin‐positive cells were more abundant in the graft than glucagon‐positive cells.^26^ Furthermore, the presence of CD31‐positive endothelial cells around coated islets indicated neovascularization of the transplant, and this presence was higher compared to non‐coated islets (1.4% vs. 1.0%), though the difference was not statistically significant (Figure 6a,b). Altogether, these findings indicated that coated islets retain secretory function and allow for vascularization around the islets post‐transplantation.

## 4 DISCUSSION

Conformal coatings have been previously described in the literature as an ideal configuration for islet encapsulation and immunoprotection.^8^, ^27^, ^28^ Aside from providing a physical barrier against immune cells and antibodies, the exact mechanisms by which conformal coatings interact with immune cells to impart immunoprotective effects on islets depend on material surface chemistry. The influence of material surface chemistry on immune cell recruitment is well described in relation to protein adsorption or macrophage polarization.^29^, ^30^ In the context of conformal coating, PEG is a common material that exhibits anti‐fouling properties by reducing protein adsorption. However, the uprise in PEG antibodies has led to the development of alternative polymers to impart anti‐fouling properties, a concept that motivated for the development of the conformal coating described in this study. We selected phosphocholine and triazole‐based modifications for our biocompatible coating composed of chitosan and alginate. A major emphasis has been given to zwitterionic groups such as phosphocholine, which have been applied as anti‐fouling polymers for a variety of applications.^31^, ^32^ Triazole‐alginates were also established as non‐fouling and anti‐fibrosis implant materials tested for immunoprotective effects in transplants.^14^ However, most investigations of these chemical groups in the context of immunoprotection are based on bulk‐material properties and not for nanocoating‐based applications. Therefore, in vitro assessment of our conformal coating provided unique insights on the interaction of such coatings with the immune system.

We have previously validated the LbL deposition and the biocompatibility of our proposed coating with PC‐QCH and TZ‐AL using MIN6 cell‐derived spheroids in vitro and in vivo.^9^ Our previous results showed reduced pro‐inflammatory cytokine production in co‐culture models using human THP‐1 and mouse RAW 264.7 macrophage cell lines. Following these results along with literature indications that our materials may affect innate immune cell recruitment or function, we sought to evaluate the effects of our coating on primary mouse‐derived splenocytes enriched for antigen presenting cells (APCs). Our results demonstrate that these materials abrogate LPS‐mediated APC activation and inflammation, with higher doses of polymers achieving a greater degree of inhibition compared to lower doses and untreated controls. We observe that the polymers inhibit the activation of APCs, particularly B cells, in a dose‐dependent manner at an optimal inflammatory dose of LPS (1 μg/mL), suggesting that these materials may promote a tolerogenic or anti‐inflammatory phenotype in APCs. Several studies point to the fact that chitosan and alginate signal through TLR4, as LPS does to mediate its inflammatory effects.^33^, ^34^, ^35^, ^36^ This raises multiple questions and possibilities, one being that these materials shift APCs toward an anti‐inflammatory phenotype by preferentially signaling through the less inflammatory, TRIF‐dependent pathway.^37^, ^38^ Additionally, it is possible that the polymers compete with LPS for TLR4 binding, especially at higher doses, which may explain why these polymers blunt APC activation marker expression in highly inflammatory conditions. Future studies will focus on whether the polymers depend on TLR4 to execute their observed effects on APCs, which will allow us to elucidate the cell‐intrinsic mechanisms driving diminished APC activation in the presence of these materials.

APC activation was also reduced in the presence of coated polystyrene microspheres compared to non‐coated microspheres. Polystyrene microspheres served as a model to isolate the coating interaction with immune cells, eliminating confounding factors present when using coated islets/cell spheroids. This approach mirrors previous studies on coating immunogenicity, such as the use of sacrificial silica microparticles to assess a conformal coating with PVPON (poly N‐vinylpyrrolidone) and tannic acid.^39^ In our study, coated microspheres mitigated APC activation similar to polymers in solution, although the coating's effects were less pronounced. This difference likely reflects the fact that a lower polymer dose is adsorbed onto microspheres compared to the higher doses of polymer solutions used for the assay presented in Figure 2. Polymer surface adsorption depends on concentration and incubation time, both of which were optimized in our previous work to minimize stress and maximize adsorption onto cell surfaces.^9^ Consequently, the effects observed with coated microspheres closely resemble those expected with coated islets, providing a valuable model for future studies on new formulations and modifications of similar coatings.

Coating properties such as thickness, permeability, and viscoelasticity were characterized in our previous study using MIN6 beta cells.^9^ Similar to our findings with MIN6‐derived spheroids, the PC‐QCH/TZ‐AL coating did not present a negative effect on GSIS or cell viability of primary mouse islets. Transplanting coated islets into syngeneic diabetic mice achieved normoglycemia, albeit with a slight delay compared to non‐coated islets, evident in the diabetes reversal rate (Figure 5c) and IPGTT blood glucose values at 45 and 60 min (Figure S5). However, IPGTT AUC, C‐peptide quantification, and insulin immunostaining showed that coating did not impair graft function or insulin production. These results indicate that coated mouse islets maintain secretory function in syngeneic transplant models, consistent with previous studies using beta cell lines and our in vitro findings in the present study. In addition, transplanted coated islets also recruited CD31‐positive endothelial cells, indicating potential vascularization, a key parameter for islet survival in vivo. The highly vascularized native islet microenvironment within the pancreas should be simulated during transplantation to provide adequate perfusion for islet function and glucose sensing.^40^ Thus, coated mouse islets retain therapeutic efficacy for glycemic control in T1D.

Syngeneic models confirm feasibility and lack of negative effects on engraftment, but allogeneic models are necessary, along with performing flow cytometry, to demonstrate efficacy regarding immunoprotection and better understand the APC activation and innate cell activity during the transplant process.^1^ Furthermore, STZ‐induced diabetic rodent models do not reflect autoimmunity, which could be addressed using syngeneic or allogeneic transplants in spontaneously diabetic NOD mice.^1^, ^8^ These limitations highlight opportunities for future research. On the other hand, flow cytometric analysis of in vitro immune cell activation in response to biomaterials is a powerful tool providing extensive information on cell viability, proliferation, phenotype, activation, and maturation. This approach allows for rapid development and optimization of novel immunomodulatory materials, offering a valuable proof‐of‐concept often lacking in the literature. Overall, these results provide a benchmark for future studies evaluating the coating's efficacy in preventing immune rejection of islet allografts. Furthermore, the coating can be investigated in a wide range of applications to limit transplant rejection, including the encapsulation of other cell types and the surface modification of solid implants to reduce immunogenicity.

## 5 CONCLUSION

Conformal coatings are an ideal configuration for encapsulation of cell transplants when implemented with materials that can impart immunomodulatory effects. This study validates the biocompatibility of our PC‐QCH/TZ‐AL nanocoating in vitro and in vivo using primary mouse islets, expanding on our previous work with coated mouse beta cell spheroids. In addition, we demonstrate through in vitro assays the immunoprotective potential of our proposed materials and nanocoating for islet encapsulation. The ability of our polyelectrolytes to attenuate APC activation supports previously reported effects of PC and TZ‐modified macromolecules in reducing innate immune cell recruitment and activation. Therefore, these materials offer promise for cell‐based therapies beyond islet encapsulation toward cell‐based therapies for other diseases.

## CONFLICT OF INTEREST STATEMENT

The authors declare no conflict of interest.

## Supporting information


**Data S1.** Supporting Information.

## Data Availability

The data are available upon request.
