# Supplementary material for: Evaluation of a functionalized chitosan and alginate multilayer conformal nanocoating toward improving islet survival in syngeneic mouse islet transplantation
Source: Bioeng Transl Med. 2026 Jan 21;11(3):e70039. doi: 10.1002/btm2.70039 (PMC13247435; doi:10.1002/btm2.70039)
Supplement: Supplementary file 1 — Data S1. Supporting Information. [file BTM2-11-e70039-s001.docx]

Supporting Information

Evaluation of a functionalized chitosan and alginate multilayer conformal nanocoating towards improving islet survival in syngeneic and allogenic mouse islet transplantation

Michael Yilma Yitayew^1^, Alexandre Bay^2-4^, Ling Li^5^, Ciriaco A. Piccirillo^2-4^, Maryam Tabrizian^1,5,6,*^

1. Department of Biomedical Engineering, McGill University, Montréal, QC, Canada

2. Department of Microbiology and Immunology, McGill University, Montreal, QC, Canada

3. Program of Infectious Diseases and Immunity in Global Health, Centre for Translation Biology (CTB), The Research Institute of the McGill University Health Centre (RI-MUHC), Montreal, Quebec, Canada

4. Centre of Excellence in Translational Immunology (CETI), McGill University, Montreal, Quebec, Canada

5. Department of Anatomy and Cell Biology, McGill University, Montréal, QC, Canada

6. Faculty of Dental Medicine and Oral Health Sciences, McGill University, Montréal, QC, Canada


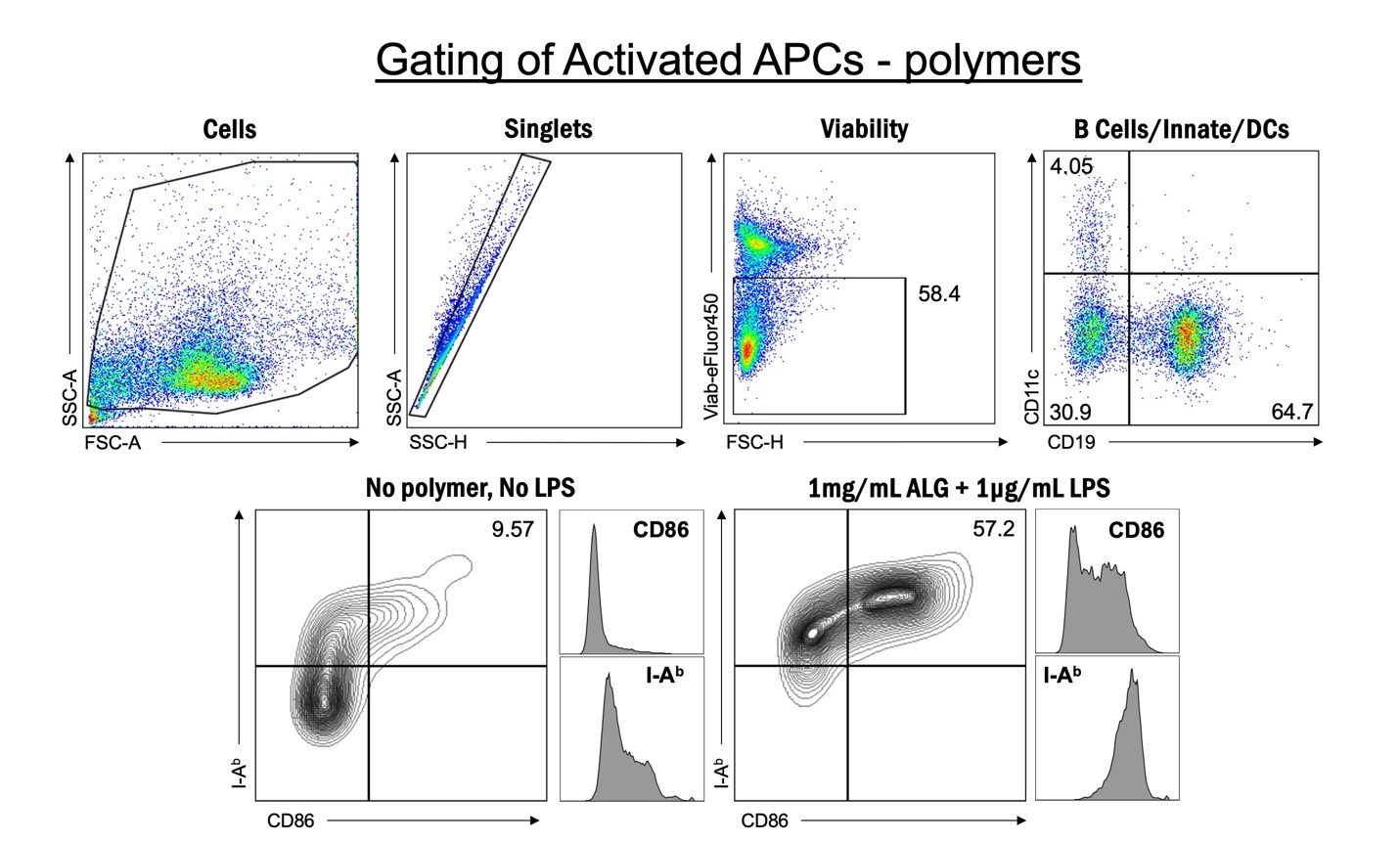

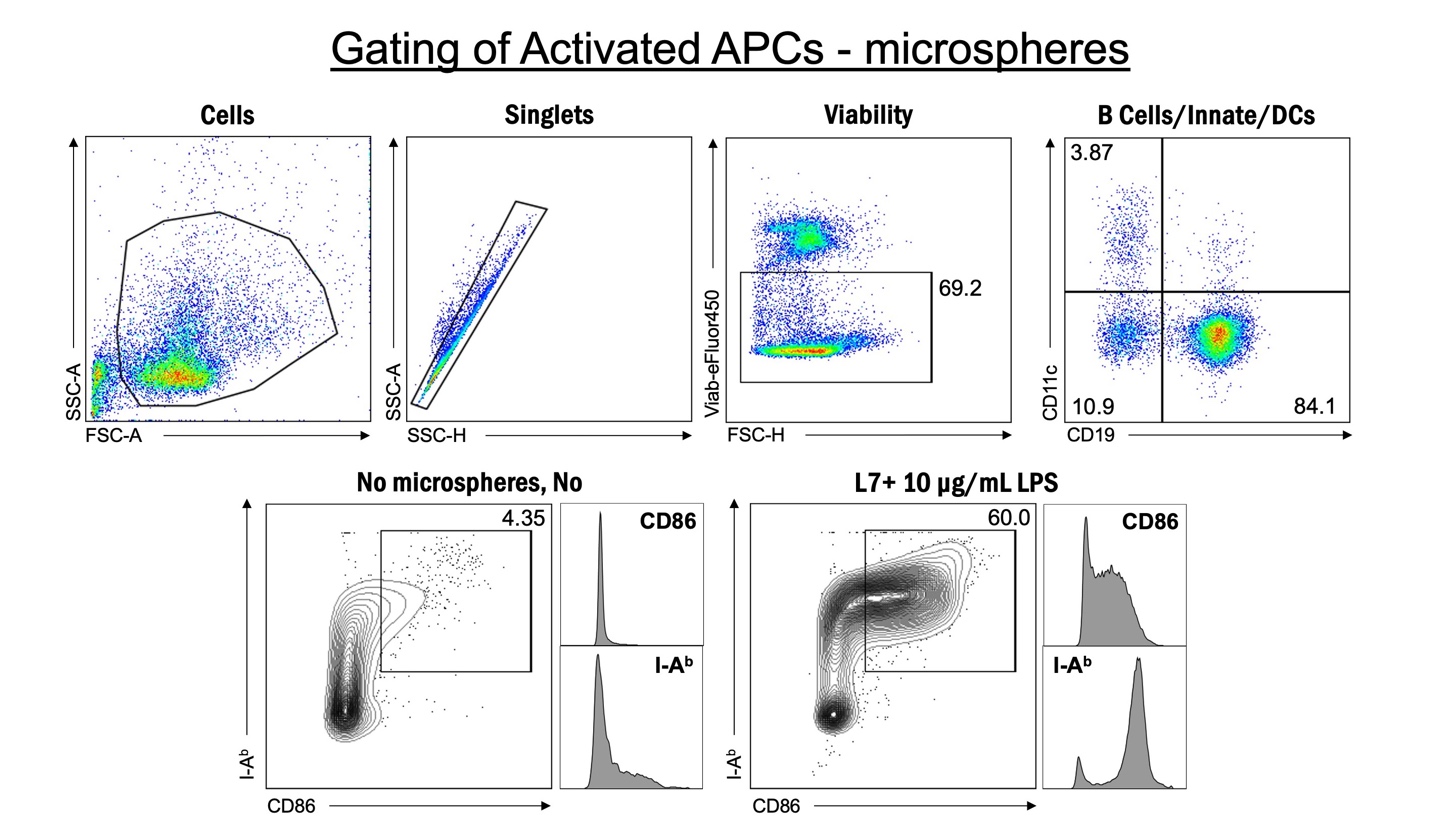


Supplemental Figure 1 – Flow cytometry gating scheme for assessing APC stimulation with different concentrations of LPS and polymer solutions (top) or coated microspheres (bottom).


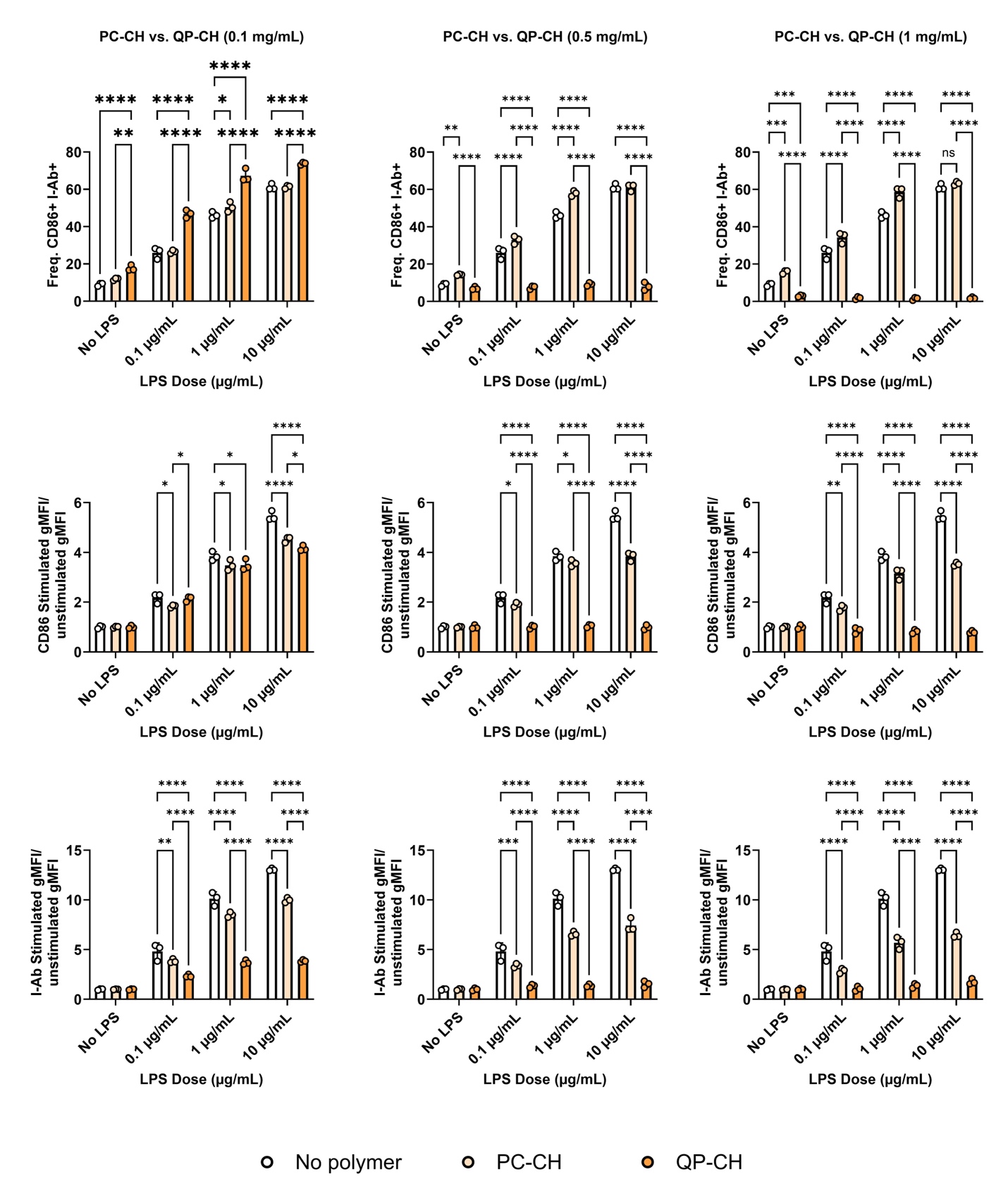


Supplemental Figure 2 - APC stimulation with different concentrations of LPS and PC-CH vs. PC-QCH followed by flow cytometry analysis of CD86 and I-Ab+ cell frequency. gMFI of CD68 and I-Ab are represented as fold change relative to cells with no LPS. Data represented as mean ± SD. ns = P > 0.05, * P ≤ 0.05, ** P ≤ 0.01, *** P ≤ 0.001, **** P ≤ 0.0001, two-way ANOVA with Tukey’s test.


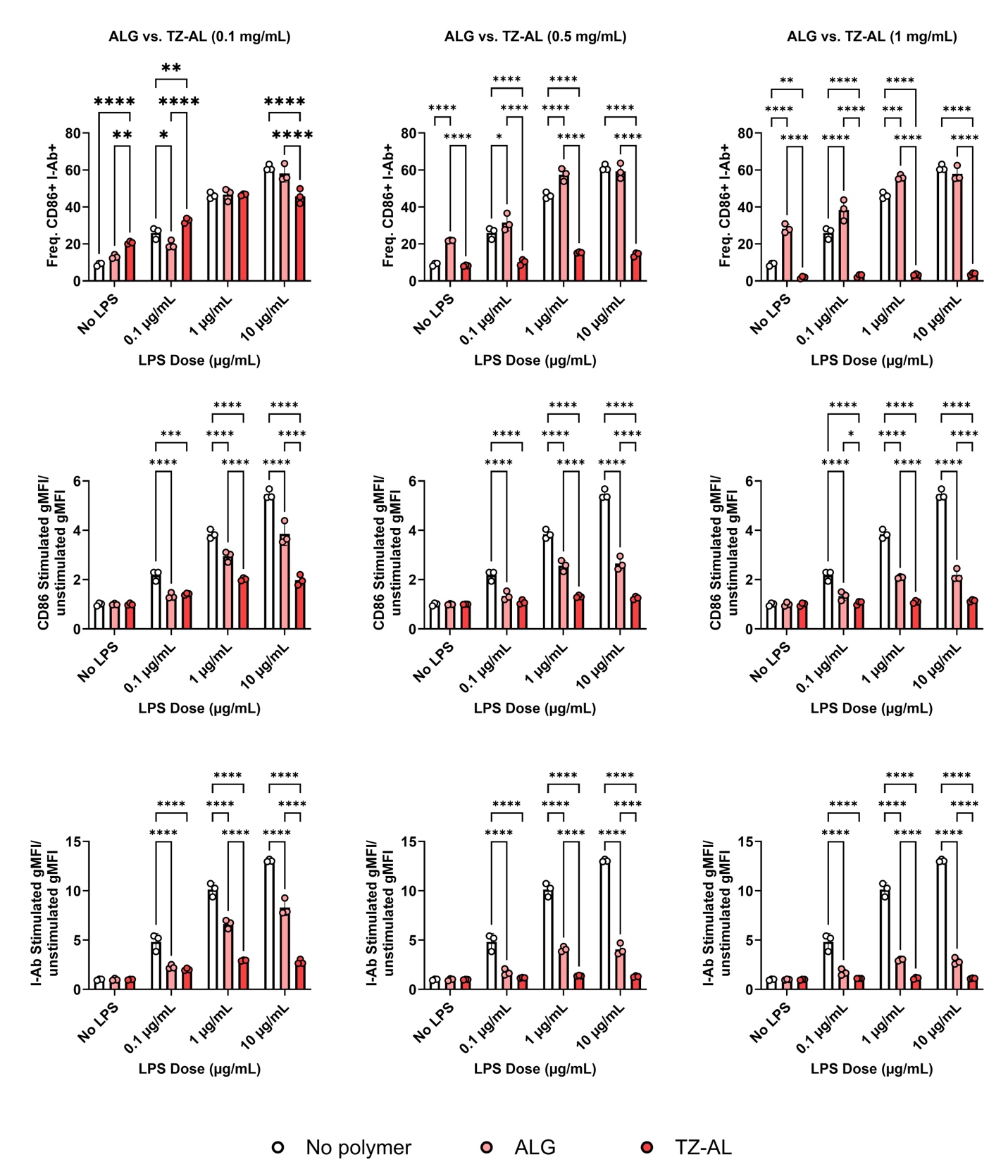


Supplemental Figure 3 - APC stimulation with different concentrations of LPS and ALG vs. TZ-AL followed by flow cytometry analysis of CD86 and I-Ab+ cell frequency. gMFI of CD68 and I-Ab are represented as fold change relative to cells with no LPS. Data represented as mean ± SD. ns = P > 0.05, * P ≤ 0.05, ** P ≤ 0.01, *** P ≤ 0.001, **** P ≤ 0.0001, two-way ANOVA with Tukey’s test.


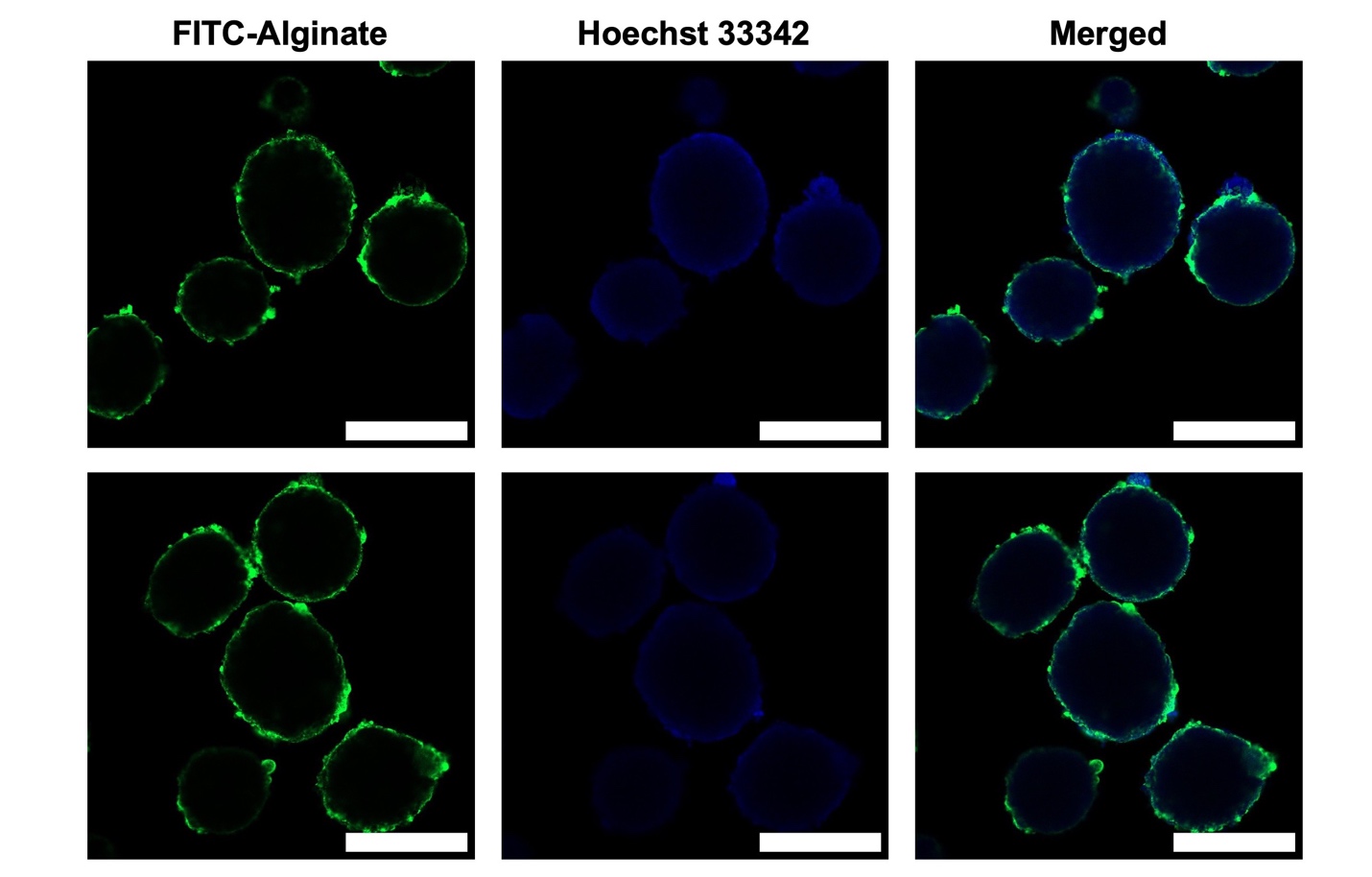


Supplemental Figure 4 - Confocal microscopy images of coated mouse islets with FITC-labeled alginate (green) and Hoechst 33342 nuclear dye (blue) (A). Scale bar = 200 μm.


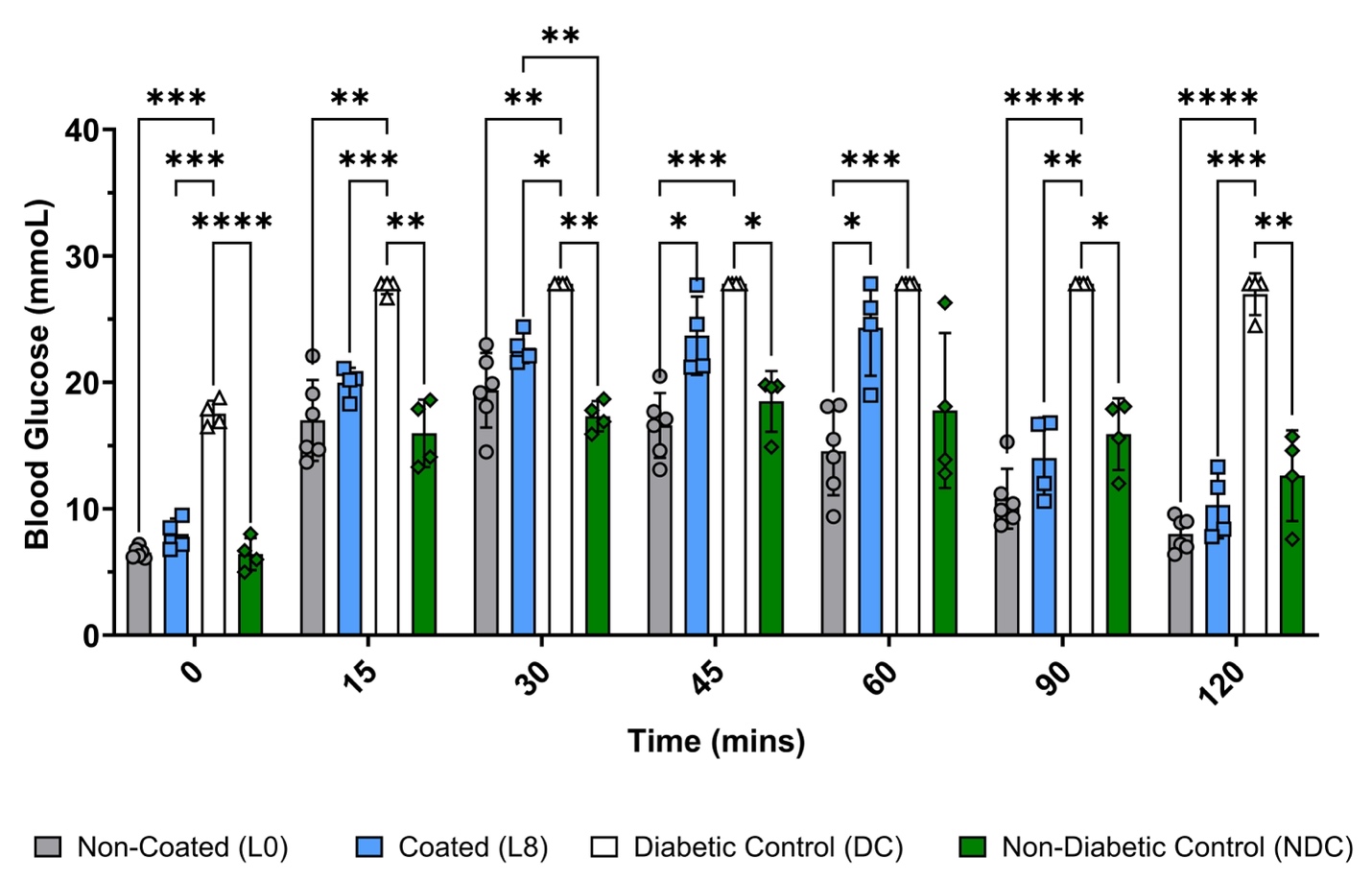


Supplemental Figure 5 – Comparison of individual timepoints for intraperitoneal glucose tolerance test (IPGTT) 30 days after kidney sub-capsular transplantation (n=6 non-coated, n=4 coated, n=4 diabetic control (DC), and n=4 non-diabetic control (NDC)). Data represented as mean ± SD. * P ≤ 0.05, ** P ≤ 0.01, *** P ≤ 0.001, **** P ≤ 0.0001.
